# Co-producing Progression Criteria for Feasibility Studies: A Partnership between Patient Contributors, Clinicians and Researchers

**DOI:** 10.3390/ijerph16193756

**Published:** 2019-10-06

**Authors:** Hannah M. L. Young, Samantha Goodliffe, Meeta Madhani, Kay Phelps, Emma Regen, Anthony Locke, James O. Burton, Sally J. Singh, Alice C. Smith, Simon Conroy

**Affiliations:** 1 Department of Respiratory Sciences, University of Leicester, Leicester LE1 7RH, UK; 2 John Walls Renal Unit, University Hospitals of Leicester NHS Trust, Leicester LE1 5WW, UK; 3 Leicester Kidney Lifestyle Haemodialysis Patient Involvement Group, University of Leicester and University Hospitals of Leicester NHS Trust, Leicester LE1 5WW, UK; 4 Department of Health Sciences, University of Leicester, Leicester LE1 7RH, UK; 5 Aging Related Research Patient and Public Involvement Group, University of Leicester, Leicester LE1 7RH, UK; 6 National Centre for Sport and Exercise Medicine, Loughborough University, Loughborough LE11 3TU, UK; 7 Department of Cardiovascular Science, University of Leicester, Leicester LE1 7RH, UK; 8 Department of Respiratory Medicine, Glenfield Hospital, University Hospitals of Leicester, Leicester LE1 5WW, UK

**Keywords:** feasibility, progression criteria, co-production, patient involvement, consensus, nominal group technique

## Abstract

There is a lack of guidance for developing progression criteria (PC) within feasibility studies. We describe a process for co-producing PC for an ongoing feasibility study. Patient contributors, clinicians and researchers participated in discussions facilitated using the modified Nominal Group Technique (NGT). Stage one involved individual discussion groups used to develop and rank PC for aspects of the trial key to feasibility. A second stage involving representatives from each of the individual groups then discussed and ranked these PC. The highest ranking PC became the criteria used. At each stage all members were provided with a brief education session to aid understanding and decision-making. Fifty members (15 (29%) patients, 13 (25%) researchers and 24 (46%) clinicians) were involved in eight initial groups, and eight (two (25%) patients, five (62%) clinicians, one (13%) researcher) in one final group. PC relating to eligibility, recruitment, intervention and outcome acceptability and loss to follow-up were co-produced. Groups highlighted numerous means of adapting intervention and trial procedures should ‘change’ criteria be met. Modified NGT enabled the equal inclusion of patients, clinician and researcher in the co-production of PC. The structure and processes provided a transparent mechanism for setting PC that could be replicated in other feasibility studies.

## 1. Introduction

Complex health interventions involve multiple context-dependent and inter-acting components [1,2]. Consequently, they can be challenging to evaluate and implement. Feasibility studies explore the viability of a definitive randomised controlled trial (RCT) for complex interventions [2,3]. These types of studies are advocated when addressing key uncertainties around the design of the trial and intervention, with the overall aim of deciding whether, and how, to proceed to a full scale RCT [3,4,5,6,7]. Judgement regarding this is based upon a set of ‘progression criteria’ that are established a priori to facilitate decision-making [3,5,6].

Existing guidance has broadly identified the key areas of an RCT that require progression criteria [8]. For each criterion, the development of ‘stop’, ‘change‘ and ‘go’ thresholds is recommended, and these are typically expressed as a percentage [9]. The ‘stop’ thresholds indicate when there are issues that cannot be resolved, and ‘go’ thresholds when there are no issues that may impede the success of a trial. The ‘change’ threshold allows researchers to identify where there are issues that may be remedied. Enabling modification may then render a definitive RCT viable [5,9]. A systematic process for the application of these progression criteria following the completion of a feasibility study has been outlined [1], but a recent review by Hallingberg et al [7] highlighted a lack of guidance on how to devise specific a priori progression criteria [7,9]. Given this lack of guidance progression criteria are decided on an ad hoc basis, in response to the specific focus of the research, nature of the intervention and the context in which it will be applied [10]. 

Patient and public involvement (PPI), defined as “research being carried out ‘with’ or ‘by’ members of the public rather than ‘to’, ‘about’ or ‘for’ them” [11] has been shown to improve the validity, quality and relevance of feasibility studies [12]. The active participation of patient and clinician contributors is crucial to the development of progression criteria. The feasibility of future clinical implementation is as important as the feasibility of future efficacy testing [1,7,13,14,15]. Thus, experience of living with a health condition, and balancing trial feasibility with future ‘real-world’ effectiveness need to be considered alongside trial design, the nature of the intervention studied and the characteristics of the population of interest, in order to develop meaningful progression criteria [1,7,11]. Typically, the task of deciding progression criteria is undertaken by trial steering committees (TSCs) [3,9,16,17]. The involvement of lay members and clinicians within these committees is often optional or limited to consultation [12,18,19,20]. The methods used for developing progression criteria, and any attempts to include the differing perspectives of patients and clinicians are also rarely described.

We aimed to develop a robust and transparent method for co-producing progression criteria for frailty, falls and the role of exercise in haemodialysis patients: a mixed-methods randomised controlled feasibility study (the FLEX-HD study), which was equally inclusive of patient contributors, clinicians and researchers. The primary aims of the FLEX-HD study (ISRCTN12840463) are: to determine whether an RCT investigating the effects of an exercise intervention is feasible in people living with frailty and receiving haemodialysis, and to identify how the intervention may be tailored to the needs of this group.

There is an increasing body of literature on involving PPI members in trial design, but relatively few that specifically explore methods to share ownership around methodological or analytical decision-making [19]. The aims of this manuscript are to: outline the process used to co-produce progression criteria for a feasibility study of a complex health intervention; to describe how patient contributors, clinicians and researchers were involved as equal partners within this process; and to illustrate the use of the process using the FLEX-HD study as an example.

## 2. Materials and Methods 

### 2.1. Design 

Progression criteria were developed using the Nominal Group Technique (NGT), which is a structured, systematic, transparent and inclusive method for both flexibly generating ideas, and quickly coming to consensus in a face-to-face environment [21,22]. NGT involves: asking the group members to silently come up with ideas related to the given topic of discussion; sharing them; carrying out a group discussion; and finally voting and ranking them [22,23]. These components help facilitate the equal participation of all group members, promoting the sharing of power and the ability for all to take a key role [14,21,22,23].

**Figure 1:**
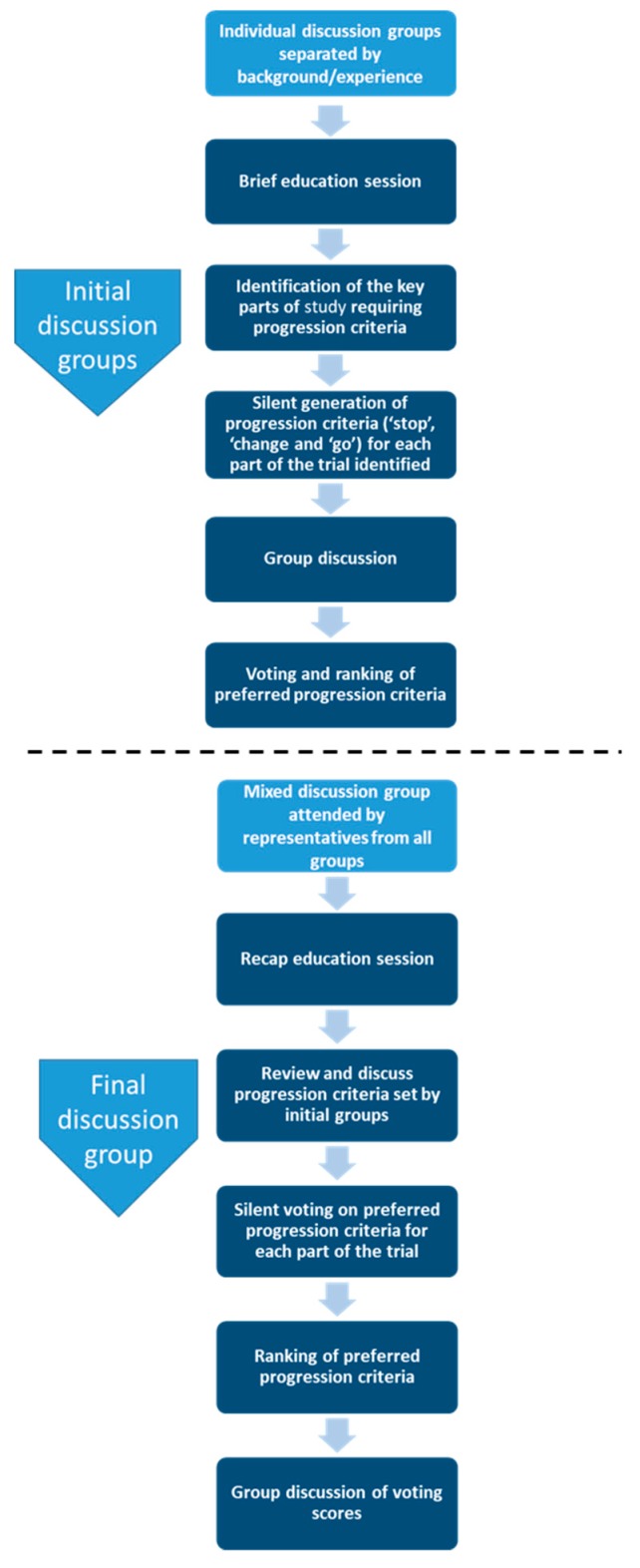
Adapted Nominal Group Technique Method used to establish ‘stop’, ‘change’ and go progression criteria.

The method can be adapted for a range of purposes [22,24,25,26]. We introduced a two-stage process (Figure 1) that facilitated the inclusion of a greater number of stakeholders and allowed us to provide brief face-to-face education sessions that provided group members with sufficient background information to participate equally and allowed them to seek further clarification throughout the process if required. 

### 2.2. Group Members and Settings 

Potential patient contributors were identified through pre-existing patient and public involvement (PPI) groups within the University of Leicester and University Hospitals of Leicester NHS Trust, and invited to be involved based on their experience of living with frailty and receiving haemodialysis. Clinicians and researchers were identified via contacts within the University of Leicester, University Hospitals of Leicester NHS Trust and Lancashire Teaching Hospitals NHS Foundation Trust, and invited based upon experience related to renal disease, frailty or exercise provision, or because they represented professions most likely to be involved in delivering a definitive RCT or implementing the final intervention within clinical practice in the future. 

Groups were held at the University of Leicester, Leicester General Hospital and Royal Preston Hospital at a time convenient to members. Patient contributors were reimbursed for their time and travel, in line with best practice guidance from INVOLVE, a national advisory group within the UK that aims to support active patient and public involvement in NHS, public health and social care research [27]. They lasted 60–90 minutes and were moderated by two facilitators (HY and SG), one of whom (HY) was involved in the FLEX-HD study. Moderators aimed to have minimal influence at all stages [28,29]. Group discussions were audio-recorded to provide an *aide memoire* to the research team. Verbal consent for the discussions to be audio-recorded was sought, but recordings were neither transcribed nor analysed [30]. The proformas used for developing the progression criteria and voting (described in Section 2.3 and Section 2.4) formed the basis of the results described.

### 2.3. Stage One. Initial Discussion Groups 

Plain English written materials, including an outline of the purpose and procedures used within the groups and a glossary of terms, were given to members before group attendance [31]. Prior understanding was not assumed, and all groups were provided with the same information. Greater explanations of medical terminology were provided to patient contributors and the language used adapted in consideration of possible negative connotations surrounding the term ‘frailty’ [32]. Large print versions of the materials were produced for visually impaired members.

Members of the initial discussion groups were grouped based upon whether they were a clinician, researcher or patient contributor to promote free discussion. Sessions began with a brief education session that provided the background information necessary to actively contribute [33]. The content of the session is outlined in Table 1. The written materials and the content of the education sessions were developed by researchers (HY, SG, KP, ER), and a PPI member with experience in health research and education (MM). 

**Figure 2:**
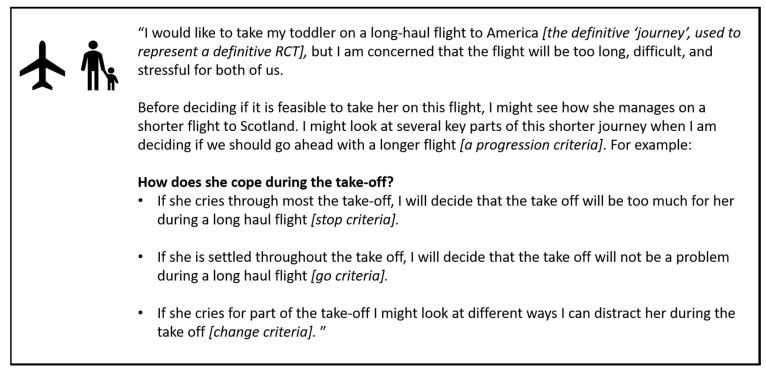
Example analogy to explain progression criteria and how ’stop’, ‘change’ and ‘go’ thresholds might be applied to aid decision-making.

Patient and clinician groups were also provided with an everyday analogy to facilitate their understanding of progression criteria (Figure 2) [9]. As most groups had a variety of experience of research and trials, following the education session, the key aspects of the feasibility study that typically require progression criteria were outlined by the group moderators [5,8]. These were eligibility and recruitment rates, intervention and outcome acceptability, and loss to follow-up. These terms are ill-defined within the literature [7], and therefore groups were also provided with plain English definitions of these concepts, which are outlined in Table 2. Group members were also invited to suggest any additional areas of the study where progression criteria may be indicated specifically for the FLEX-HD study. 

**Figure 3:**
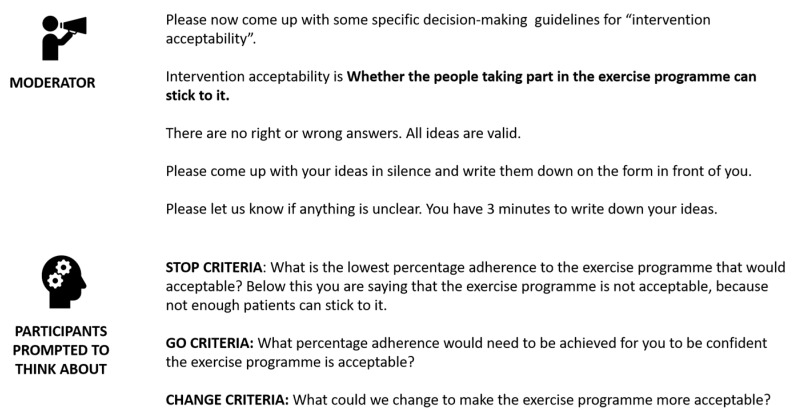
Example of how group members were prompt to consider the progression criteria, using intervention acceptability as an example.

Members were then asked to suggest progression criteria for each of these aspects of the study, in turn. They were prompted to consider these using three basic questions relating to the ‘stop’, ‘change’ and ‘go’ criteria, which were adapted according to the specific aspect of the trial being addressed. An example is provided within Figure 3. 

For each aspect of the trial, members were provided with a proforma to document their ideas for progression criteria for both ‘stop’ and ‘go’ thresholds, expressed as a percentage [22]. They were also informed that the ‘change’ criteria would fall between the ‘stop’ and ‘go’ thresholds and asked to document what changes could be made that might increase feasibility or acceptability. Supplementary Materials Table S1 provides a generic proforma that may be adapted for use in other studies. Members had three minutes to complete the task for each aspect of the trial. [22,23]. Typically, NGT requires participants to generate ideas for only one or two questions, but as our process required at least five aspects of the trial to be considered, the generation of ideas was time-limited [23].

No discussion was allowed, but clarification could be sought, if required [22]. All the progression criteria were collated, discussed and combined where appropriate [23]. In accordance with conventional NGT methodology, the intention was for the groups to then rank the progression criteria for each part of the trial in order of preference, to arrive at a set of final ‘preferred’ progression criteria [22].

### 2.4. Stage Two. Final Discussion Group 

The aim of the final discussion group was to bring together contributors of all backgrounds to review the progression criteria generated by the initial groups and decide upon the final preferred progression criteria [34]. Members of the mixed final group were asked not to discuss their backgrounds to reduce potential power differentials [29]. The education session was repeated, and the anonymised progression criteria developed by the initial groups presented. Instances where multiple initial groups had come up with the same progression criteria were highlighted [25]. Fifteen minutes was allocated for group members to review and refine these until there was a condensed list. Group members were then asked to vote for their ‘top three’ preferred criteria from this list, using a proforma. A generic version of this proforma is included within Supplemental Materials Table S2. Voting was completed for each aspect of the trial in turn, without conferring [23]. Members awarded their preferred option the highest score (1) and continue ranking to 3, for their least preferred option. Moderators scored the votes for each potential ‘stop’ and ‘go’ criteria. Progression criteria voted in first place were awarded a score of 3, the second a score of 2 and the last a score of 1. The highest scoring criteria became those used in the FLEX-HD study [25]. In instances where scores were tied, further discussion and an additional vote was allowed until a preferred criterion was identified [34]. All contributors were given a written summary of the findings once all the groups were completed.

### 2.5. Impact Evaluation

The specific impact of the involvement of the patient contributors was not formally evaluated. We aimed for all members to work in partnership and singling out one group to determine the impact of their involvement felt incongruous [35]. All members were, however, invited to give verbal feedback on what worked well and what could be improved in relation to the process at the end of the discussions. 

### 2.6. Ethics Approval

The UK Health Research Authority (HRA) toolkit [36] determined that the work was not considered research, as participants were not randomised, and no change to usual care or intervention was made. In addition, although the process described may be transferrable to other studies, the ‘results’ (i.e., the progression criteria) were specific to the FLEX-HD study, and were used solely to illustrate the process. Furthermore, joint guidance from the HRA and INVOLVE states that ethical approval is only required if patients are conducting research as part of the research team, or if they are involved in the study as participants [37]. In accordance with this national guidance, and following confirmation from the University of Leicester (sponsor of the FLEX-HD study), ethical approval was not required for this PPI activity.

## 3. Results

### 3.1. Initial Groups 

Eight initial groups were held, involving a total of 52 members: 15 (29%) patient contributors, 13 (25%) researchers and 24 (46%) clinicians. The characteristics of group members involved in the initial discussion groups are outlined in Table 3. The research groups included seven (54%) research associates and six (46%) clinical academics with a median of seven (interquartile range (IQR) 4–22) years’ experience. The clinician groups included nine (36%) physiotherapists, nine (36%) renal nurses, five (20%) occupational therapists and two (8%) nephrologists with a median of 14 (3–24) years’ experience. The patient contributor groups included six (40%) people living with renal disease and nine (60%) older people living with frailty. Across all the groups 37 (71%) were female; 45 (86%) were White British, six (11%) Asian British, one (1.5%) Black British and one (1.5%) did not wish to state their ethnicity. Researchers were a median age of 44 (IQR 26–48) years, clinicians a median age of 38 (IQR 33–49) and patient contributors 63 (50–82) years. In total 32 (62%) had previously been involved in research in any capacity. 

The percentage progression criteria developed for each of the aspects of the trial by each initial group are outlined in Table 4. Whilst the groups came to consensus over their preferred criteria through discussion (negating the need for the ranking task originally planned), Table 4 clearly demonstrates the wide range of progression criteria produced across the different groups. Additionally, other aspects of the trial, namely the acceptability of the randomisation procedures, intervention fidelity and incidence of adverse events and harms were identified as important by a minority of groups, reflecting the differing priorities and levels of knowledge amongst the groups. As there was uncertainty around whether these warranted progression criteria, they were not subject to voting and ranking within the final group. 

### 3.2. Characteristics of Final Groups 

The final joint discussion group involved 8 members: two (25%) patient contributors, one (13%) nephrologist, one (13%) researcher, two (25%) physiotherapists and two (25%) occupational therapists. Four (50%) were female, five (63%) were White British and three (38%) Asian British, with a median age of 45 (IQR 29–64) years. Six (80%) members had been involved in an initial group and four (50%) had previously been involved in research in any capacity. 

### 3.3. Ranking of Progression Criteria 

Table 5 outlines the scores given to each of the condensed progression criteria following the voting task. All the criteria ranked in first place received 67 to 100% of the votes. 

### 3.4. Final Progression Criteria 

**Figure 4:**
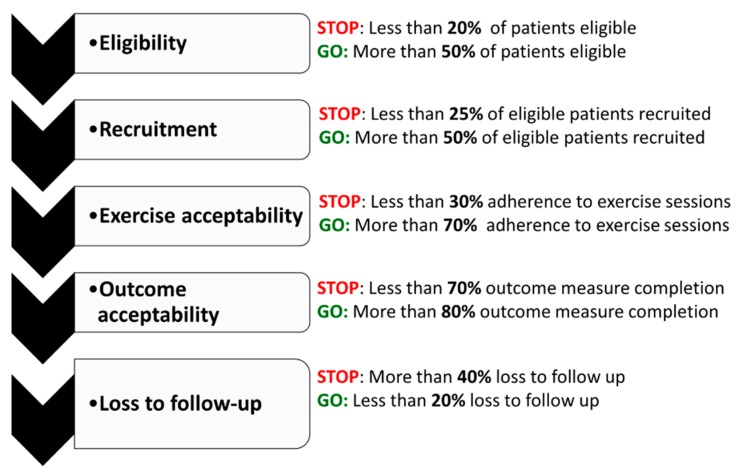
Final progression criteria developed for the FLEX-HD study.

The final preferred ‘stop’ and ‘go’ progression criteria selected are presented within Figure 4. Greater flexibility was afforded to exercise adherence to allow for periods of ill-health, hospitalisation and holidays over the duration of the intervention. The thresholds for outcome measure completion were more stringent, in recognition of the high levels of data completion required to make robust conclusions about efficacy in a definitive RCT. The potential need for different ‘stop’ and ‘go’ thresholds for different types of outcome measures was highlighted. For example, physical function measures were time-consuming, burdensome and subject to greater influence from symptoms and illness than patient-reported measures, thus requiring more flexible thresholds for completion. Four groups stated that loss to follow-up should be assessed at interim, and one group indicated that progression criteria established for loss to follow up should be applied to both the intervention and control arms of the study separately.

### 3.5. Change Criteria

**Figure 5:**
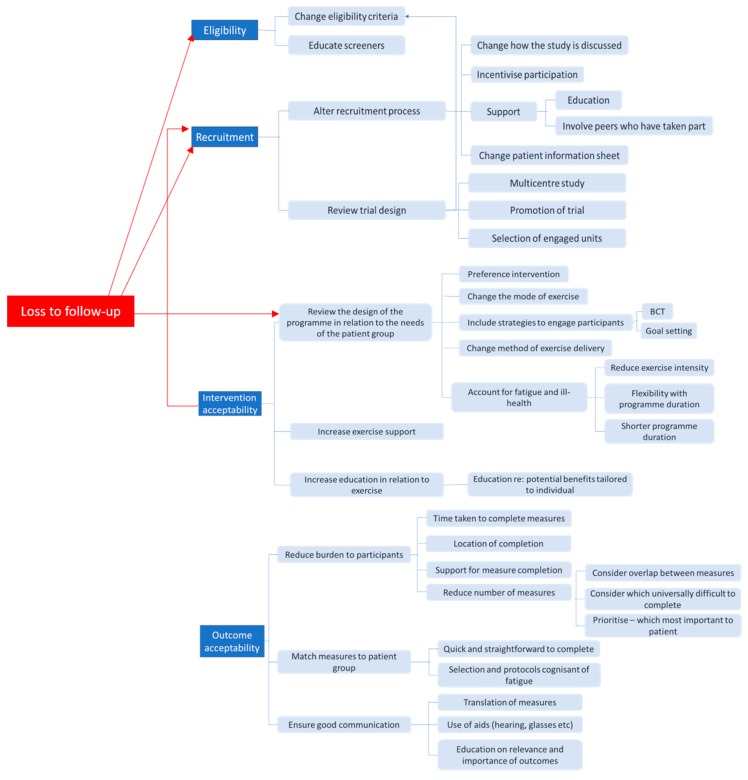
Suggestions for amendments to the trial and intervention should thresholds for ‘change’ criteria be met; BCT: Behaviour change techniques.

Patient contributors, clinicians and researchers across groups identified multiple different ways that the trial and intervention could be amended, should results fall between the ‘stop’ and ‘go’ thresholds, and indicate that ‘change’ is required. These are summarised within Figure 5.

Thirteen (25%) initial group members identified that eligibility rates could potentially be boosted by widening the eligibility criteria, particularly in relation to frailty levels. Regular contact with those screening for eligibility, reminding them about the study and checking that screening was being completed appropriately was recommended. The therapist groups recommended that eligibility criteria for the exercise intervention be revisited should the design and delivery of the programme change significantly. For example, some participants who might not be eligible to exercise at home unsupervised may be able to exercise safely within a supervised environment, and the eligibility criteria should reflect this difference.

All groups indicated that the intervention would be the primary area of the trial to review should strategies to boost recruitment be required. Simplifying the intervention by reducing the number of visits required, the length of the intervention and making it enjoyable to participate was important. 

The approach taken during recruitment was also an area highlighted for review. Explaining the study face-to-face and, where possible, exploring potential individual barriers to participation during the recruitment process was felt to be especially relevant in a population of older people living with frailty, whose participation may be limited by external constraints such as reliance upon packages of care and family assistance. By organising the trial procedures around these considerations, groups felt that more people may be encouraged to participate. When discussing the study, all groups also indicated that highlighting the potential benefits for the individual was important.

Involving the family in the recruitment process and enlisting the support of patient advocates and peers who had taken part in the study and giving potential participants the chance to observe the types of exercise they may be asked to participate in was also identified as important across all groups. Patient groups suggested the use of an overview booklet be used in lieu of a more traditional patient information sheet, with images alongside limited simple text. More detailed information could then be provided if interest in taking part is expressed. 

Sixteen (25%) of all members across all groups felt that varying the mode of exercise offered would increase participant engagement with the intervention by potentially making it more enjoyable. Fifteen (23%) of all participants across all groups also suggested reviewing the intensity of the exercise programme in this population. Fatigue was identified as a key factor limiting exercise adherence for people living with frailty and receiving haemodialysis, and therefore all groups recommended lowering the exercise intensity, providing participants with a longer-run in period and offering interval training (where short bouts of moderate intensity are interspersed with those of lesser intensity). Increasing the support available from exercise professionals was also identified as important. Supplementing this practical support with regular reviews to check participant progress and including techniques to support and maintain behaviour change were also suggested. 

Members postulated that reducing the number of measures used, and selecting those which were simple, quick and less intrusive but also meaningful to participants, would help to increase completion. Prioritising the measures key to the trial, conducting them at a time and location that was most convenient to the individual was also highlighted as important. Effective communication about what the measure is assessing, alongside support for outcome completion was additionally a frequently identified means of increasing outcome completion. 

It was evident during discussion that several aspects of the trial were interlinked, and that changing one part might have a positive impact on another. For example, adapting the intervention might not only increase acceptability, but might also improve recruitment and retention. Finally, all group members emphasised the importance of understanding the barriers to feasibility from the perspectives of both participants and clinicians. They deemed this crucial to identifying those amendments most likely to result in successful delivery of the future definitive trial.

### 3.6. Resource Implications

The complete process took four months (including preparation time) and a total time of 9.5 hours to complete. The total running costs for the groups (refreshments, room hire, expenses and payments to some patient contributors) was £975. 

### 3.7. Impact and Feedback 

Group members valued being actively involved in setting progression criteria that would have a direct impact upon the FLEX-HD study; however, they found the application of abstract concepts and theoretical percentages challenging. They suggested that more time be allowed, and that members of the final discussion group be given an outline of the results from the individual groups to allow greater time for reflection prior to discussion. 

## 4. Discussion

This work describes a transparent and collaborative process for developing progression criteria that are a critical part of determining whether a definitive RCT is feasible [3]. Some components were study specific, but the overall structure and process could be replicated within other feasibility studies. This represents a first step in addressing the paucity of guidance detailing how progression criteria should be developed [2,7]. A template for generating and subsequently voting on progression criteria for use in other feasibility studies is included within Supplementary Materials Tables S1 and S2.

### 4.1. Involving Patients, Clinicians and Researchers Equally in the Co-Production of Progression Criteria

The results of the initial groups highlight tensions between the priorities and perspectives amongst diverse groups of patient contributors, clinicians and researchers. Involving all stakeholders in the process recognises that what is feasible within a ‘real world’ clinical setting may be at odds with what is possible within a highly standardised research one [7,10,14,38]. These issues were considered and resolved in a transparent and equally inclusive manner, leading to the development of progression criteria that are meaningful within both settings. The inclusion of clinicians and patients was also particularly important within the context of the FLEX-HD study, as there is little prior research in a frail group in the field of renal rehabilitation to guide the development of progression criteria. As people living with frailty represent a ‘seldom heard’ group, who can be challenging to engage in research, their perspectives are crucial when determining feasibility [38,39,40] and working in collaboration facilitated a detailed understanding regarding how to adapt trial processes in a manner acceptable to this patient group, and the context in which they receive care. 

The use of co-production has been linked with the generation of multiple ideas for innovative intervention development and service improvement [41]; in keeping with this, the breadth of suggestions for amendments to the trial in the event of the ‘change’ thresholds being met strongly underlines the value of involving multiple stakeholders in the co-production of progression criteria. The combination of the group members’ different types of knowledge and experience provided numerous innovative ways in which an RCT could be adapted to increase feasibility, which moved beyond the scope of those suggested by the research groups alone. The application of these potential solutions may inform whether or not to proceed to a definitive trial and would complement the decision-making process outlined by Bugge et al. [1]. When considering these amendments, the challenge for the study team is three-fold. Firstly, which of the suggestions are most likely to enhance feasibility? One option would be to compare the suggestions made with the views of the study participants if a mixed-methodology approach has been employed. Secondly, what (and how many) changes constitute major alterations to the trial and intervention? Numerous or major alterations may indicate that further development and feasibility testing is required [2], but to date there is no guidance on what amount of change would suggest that further testing is warranted [7]. Thirdly, the research team should consider how proposed changes might impact both scientific rigour and resource requirements, which could also influence feasibility [8]. For example, if outcome completion levels fall within the ‘change’ threshold, providing participants with support is one means of increasing completion rates, but may also necessitate additional researchers blinded to intervention groups to provide this without introducing bias. 

### 4.2. The Challenges of Co-Producing Progression Criteria

Research literacy is a key component of co-production, because knowledge increases parity and participation [7,18,42,43]. Different levels of understanding were evident across all groups, and there were different levels of PPI and research participation experience amongst contributors. The ability of all these individuals to articulate their ideas and contribute within the group setting demonstrates inclusivity. However, there were some challenges to ensuring that all felt able to do this, and to focus those more experienced members away from issues less pertinent to feasibility studies (e.g., the need for statistical power). Whilst the education session offered aimed to ensure that all involved could contribute equally, this needed to be balanced with not overburdening group members or removing their lived or clinical experience from the forefront of their decision-making [11,19,44,45]. Similar challenges have been identified within research literacy training offered as part of community-based participatory research [46,47,48]. Evaluations of these programmes conclude that theoretical constructs may best be explained through practical examples [46]. A strength of our approach was introduction of a brief education session, which was developed with PPI members to include everyday analogies and jargon-free content to facilitate understanding [44,49,50]. Despite this, some members found it difficult to fully comprehend the abstract concept of a percentage-based progression criteria, which may, in part, be reflected in the wide range of criteria suggested in the initial groups. Visual methods of learning are an accessible and engaging way of increasing comprehension and encouraging creative thinking [41,50,51]. The use of simple graphical representations (e.g., pie charts using Microsoft Excel), or physical counters representing participant numbers, used during the groups, may have enhanced understanding of the impact of one progression criteria over other aspects of the trial. Members also fed back that additional time would be beneficial in future groups, and this may also have supported the use of visual representations. 

### 4.3. Limitations

Whilst flexibility is a key feature of the NGT method, the modifications made may have influenced the criteria obtained. The omission of the ranking stage in the initial groups occurred iteratively and may have made it more difficult for some members to express their views. However, this process was carefully moderated and occurred in groups separated by background, minimising the potential impact. Some level of subjectivity is unavoidable in consensus methodology, and the inclusion of different members or additional groups may have resulted in different criteria [33,52]. The outcomes of this process are not generalisable, nor are they intended to be, as they have been specifically developed for the FLEX-HD study. Consensus methods are grounded within a pragmatic constructivist philosophy and therefore we seek to illustrate the rigour of the process used [53].

Finally, given the resource implications outlined, and the two-stage process used, the process may be challenging to incorporate into conventional TSC structures. Patient representation on TSCs is usually limited, and there is a perceived limited impact of patient representation, questioning whether the co-production of progression criteria is possible in this setting [7,12,18,19,20]. More recently there has been a shift towards involving patient advisory panels, comprising both ‘expert’ patient contributors as well as less experienced lay members, in specific tasks responsive to the needs of the trial. Wide representation is important from all stakeholders in the development of progression criteria [20,45,54] and therefore the development of progression criteria might sit better with a patient advisory panel, alongside the research team and involved clinicians. The process could be streamlined, and resource implications reduced, by completing fewer initial groups, or by having only one mixed group inclusive of greater numbers complete both stages of the consensus processes in one sitting.

## 5. Conclusions

The use of modified NGT within diverse, structured discussion groups enabled the perspectives of patients, clinicians and researchers to be included in the co-production of progression criteria that reflected both trial and ‘real-world’ feasibility. Importantly, the process also allowed for the identification of numerous areas where the trial could be amended to increase feasibility if required. The main challenge to the process was reconciling different levels of knowledge and understanding amongst the contributors. Future groups would benefit from incorporating creative interactive and visual methods to facilitate understanding. The structure and processes used provide a transparent and flexible means of setting progression criteria that could be simplified, and replicated for use in other studies, with some adaptation to the address the specific focus of the trial required.

## Figures and Tables

**Table 1:** Outline of education session content for the initial discussion groups. RCT: randomised controlled trial.

Topic	Content
Overview of RCT design	What is an RCT? What are complex interventions? Challenges to RCTS of complex interventions
Introduction to feasibility studies	What is a feasibility study?
Introduction to progression criteria	What are progression criteria? What are they for? How are progression criteria structured? (introduction to ‘stop/go/change’ thresholds) Examples of progression criteria from other studies and analogies from everyday life
Overview of the FLEX-HD study	Aims of the study Design of the study and secondary outcomes Overview of the exercise intervention

**Table 2:** Areas requiring progression criteria, and plain English explanations provided for each during the groups.

Aspect of the Trial Requiring Progression Criteria	Plain English Explanation Provided in the Discussion Session
Eligibility	The number of patients who can take part in the study, whether they later agree to or not.
Recruitment	The number of patients who agree to take part in the study
Intervention acceptability	Whether participants can stick to the exercise programme
Outcome acceptability	Whether participants can complete the assessments at the start and the end of the study. These assessments can tell us whether intervention might be beneficial.
Loss to follow-up	The numbers of participants who drop out or were ‘lost’ (lost to follow-up)

**Table 3:** Characteristics of members of initial discussion groups. Median and IQR (interquartile range) are reported for age.

Group (n)	Members (n)	Age (years)	Gender		Ethnicity				Research Experience (yes)
			Female	Male	White British	Asian British	Black British	Not Stated	
**Researcher (2)**	13	44 (26–48)	7 (54%)	6 (46%)	10 (77%)	3 (23%)	0 (0%)	0 (0%)	13 (100%)
**Clinician (3)**	24	38 (33–49)	20 (80%)	5 (20%)	23 (92%)	1 (4%)	1 (4%)	0 (0%)	7 (28%)
**Patient (3)**	15	63 (50–82)	10 (67%)	5 (33%)	12 (80%)	2 (13%)	0 (0%)	1 (7%)	12 (80%)

**Table 4:** “Stop” and “Go” progression criteria decided by individual discussion groups. Progression criteria are expressed as a percentage; blank fields indicate that the group did not set progression criteria for that aspect of the trial.

Aspect of Trial	Group									
	Progression Criteria	Patient Groups			Researcher Groups		Clinician Groups			
		Older People’s Group	Renal Patient Group 1	Renal Patient Group 2	Age and Aging Research group	Renal Exercise and Rehabilitation Research Group		Renal and Falls Therapist Group	Older People’s Therapist Group	Renal Doctors and Nurses’ Group
		n = 8	n = 4	n = 2	n = 8	n = 5		n = 7	n = 6	n = 12
Eligibility %	Stop	<50%	<30%	<50%	<15%	<20%		<45%	<15%	<5%
	Go	>75%	>45%		>25%	>40%		>65%	>30%	>20%
Recruitment %	Stop	<25%	<35%		<30%	<30%		<20%	<25%	<40%
	Go	>80%	>60%	>20%	>30%	>50%		>55%	>50%	>50%
Intervention acceptability (Adherence %)	Stop	<60%	<50%		<25%	<65%		<65%	<50%	<50%
	Go	>75%	>60%	>40%	>75%	>80%		>70%	>75%	>70%
Outcome acceptability (Completion %)	Stop		<55%		<60%	<70–80%		<80%	<60%	<80%
	Go		>66%	>80%	>40%	>80–90%			>60%	>90%
Loss to follow up (% withdrawn or lost)	Stop	>55%	>60%	>80%	>80%	>40%		>50%	>40%	>30%
	Go	<25%	<50%	<25%	<40%	<25%		<20%	<25%	<20%

**Table 5:** Scores and ranking of condensed progression criteria. Progression criteria are expressed as a percentage. A maximum score of 24 was possible during the voting task.

Aspect of Trial	Results Agreed For Voting		Voting Scores	Ranking	Final Criteria If Tied Ranking
Eligibility	Stop	<20%	22	1	
		<30%	18	2	
	Go	>40%	15	= 2	
		>45%	15	= 2	
		>50%	16	1	
Recruitment	Stop	<20%	16	2	
		<25%	17	= 1	<25
		<30%	17	= 1	
	Go	>50%	24	1	
Intervention acceptability (adherence %)	Stop	<25%	6	3	
		<30%	21	1	
		<50%	16	2	
	Go	>70%	18	1	
		>75%	15	2	
		>80%	12	3	
Outcome acceptability (measure completion %)	Stop	<60%	19	= 1	<70
		<80%	19	= 1	
	Go	>80%	20	1	
		>90%	15	2	
Loss to follow up	Stop	>30%	17	= 1	>40
		>50%	17	= 1	
	Go	<20%	18	= 1	<20
		<25%	18	= 1	

## Supplementary Materials

The following are available online at https://www.mdpi.com/1660-4601/16/19/3756/s1. Table S1: Template for co-producing progression criteria for exploratory studies: generation of progression criteria, Table S2: Template for co-producing progression criteria for exploratory studies: voting form.
