# Supplementary material for: Co-producing Progression Criteria for Feasibility Studies: A Partnership between Patient Contributors, Clinicians and Researchers
Source: Int J Environ Res Public Health. 2019 Oct 6;16(19):3756. doi: 10.3390/ijerph16193756 (PMC6801439; doi:10.3390/ijerph16193756)
Supplement: Supplementary file 1 [file ijerph-16-03756-s001.pdf]

Supplemental Material

Table S1. Template for co-producing progression criteria for exploratory studies: generation of progression criteria.

Aspect of the Trial with Plain English Explanation	Progression Criteria and Plain English Explanations	Please Write Your Ideas Here
Eligibility: The number of patients who can take part in the study.	STOP: What is the lowest percentage of people that would need to be able to take part, whether they later agree to or not?<br>Below this percentage you think a bigger study is not possible	
	CHANGE: What could we change to increase the numbers of people that are able to take part?	
	GO: What percentage of people would need to be eligible for you to be confident a bigger study is possible?	
Recruitment: The number of patients who agree to take part in the study.	STOP: What is the lowest percentage of people that would need to take part in the study?<br>Below this percentage you think a bigger study is not possible	
	CHANGE: What could we change to encourage more people to take part?	
	GO: What percentage of people would need to take part for you to be confident a bigger study is possible?	
Intervention acceptability: Whether participants can stick to the intervention.	STOP: What is the lowest percentage of adherence to the intervention that would be acceptable?<br>Below this percentage you are saying that the intervention is not acceptable, because people are not able to stick to it enough.	
	CHANGE: What could we change to make the intervention more acceptable?	
	GO: What percentage of adherence would need to be achieved for you to be confident the intervention is acceptable?	
Outcome acceptability: Whether participants can complete the assessments at the start and the end of the study.<br>These assessments are used in the bigger study tell us whether intervention is beneficial.	STOP: What is the lowest percentage of people that would need to complete an outcome measure?<br>Below this percentage you are saying that the outcome measure isn't suitable because not enough people are completing it.	
	CHANGE: What could we change to make it easier for people to complete the measure?	
	GO: What percentage of people would need to complete an outcome measure for you to be confident a bigger study is possible?	

Aspect of the Trial with Plain English Explanation	Progression Criteria and Plain English Explanations	Please Write Your Ideas Here
Loss to follow-up: The numbers of participants who drop out or were 'lost' to follow-up.	**STOP:** What is the highest percentage of people dropping- out that would be ok?<br>Above this percentage you are saying a bigger study would not work because too many people drop out.	
	**CHANGE:** What could we change to help people stay in the study?	
	**GO:** What percentage of people dropping- out would be ok for you to still be confident a bigger study is possible?	
	**STOP:**	
	**CHANGE:**	
	**GO:**	
	**STOP:**	
	**CHANGE:**	
	**GO:**	

Notes on use: This template can be used with groups, alongside explanations from the moderator, to help them generate ideas for progression criteria in the first stage of the process. Aspects of trial design considered universally key to feasibility are included, but explanations and details can be adapted and amended as required for the specific study. Blank spaces are left for the inclusion of additional, study specific progression criteria, if indicated. Group members should be asked to document their ideas for 'stop' and 'go' criteria (which are percentage based) and suggest ideas for how the study or intervention might be changed, should results from the study fall between the stop and go thresholds. For progression criteria related to outcome acceptability there is no need to apply these to all secondary measures studied, but different progression criteria for objective and patient-reported measures could be consider.

Table S2. Template for co-producing progression criteria for exploratory studies: voting form.

Aspect of the trial and plain English explanation	Voting		Please indicate the progression criteria you are selecting below
Eligibility: The number of patients who can take part in the study.	1	Preferred/ favourite	
	2	Second favourite	
	3	Third favourite	
Recruitment: The number of patients who agree to take part in the study.	1	Preferred/ favourite	
	2	Second favourite	
	3	Third favourite	
Intervention acceptability: Whether participants can stick to the intervention.	1	Preferred/ favourite	
	2	Second favourite	
	3	Third favourite	
Outcome acceptability: Whether participants can complete the assessments at the start and the end of the study.<br>These assessments are used in the bigger study tell us whether intervention is beneficial.	1	Preferred/ favourite	
	2	Second favourite	
	3	Third favourite	
Loss to follow-up: The numbers of participants who drop out or were 'lost' to follow-up.	1	Preferred/ favourite	
	2	Second favourite	
	3	Third favourite	
	1	Preferred/ favourite	
	2	Second favourite	
	3	Third favourite	
	1	Preferred/ favourite	
	2	Second favourite	
	3	Third favourite	

Notes on use: This template can be used with groups, alongside explanations from the moderator, to vote for progression criteria in the second stage of the process. Aspects of trial design considered universally key to feasibility are included, but explanations and details can be adapted and amended as required for the specific study. Blank spaces are left for the inclusion of additional, study specific progression criteria, if indicated.
